# Squamous Cell Carcinoma Arising in a Mature Cystic Teratoma

**DOI:** 10.1155/2012/314535

**Published:** 2012-12-23

**Authors:** Suna Avcı, Fatih Selcukbiricik, Ahmet Bilici, Gülkan Özkan, Ayse Ayşim Özağarı, Fatih Borlu

**Affiliations:** ^1^Division of Internal Medicine, Sisli Education and Research Hospital, 34200 Istanbul, Turkey; ^2^Division of Medical Oncology, Department of Internal Medicine, Sisli Education and Research Hospital, 34200 Istanbul, Turkey; ^3^Division of Pathology, Sisli Education and Research Hospital, 34200 Istanbul, Turkey

## Abstract

*Introduction*. Malignant transformation in a mature cystic teratoma of the ovary is a rare complication. Squamous cell carcinoma is the most common transformation. We describe a new case of squamous cell carcinoma arising in a mature cystic teratoma. *Case Report*. A premenopausal 52-year-old female patient is diagnosed with vaginal bleeding. According to examination made on the women and the pelvic scanning, 7 cm mass is found on the right adnexa of the patient. Total abdominal hysterectomy, bilateral salpingo-oophorectomy, omentectomy, pelvic lymph node dissection, and debulking were the treatments completed on the patient. According to histopathological diagnosis, squamous cell carcinoma arising in a mature cystic teratoma is diagnosed as a reason for the mass in the right adnexa of the patient. *Conclusion*. The prognosis of the malign transformation of MCT depends on surgery stage; however it is extremely poor. The patient should receive chemotherapy regardless of stage. We have decided to administer second cycle carboplatin and paclitaxel treatments on the patient.

## 1. Introduction

Mature cystic teratoma (MCT) is the most common germ cell tumor of the ovary. MCT composes 10–20% of all ovarian tumors. MCT diagnosed as bilateral is roughly 9–16%. MCT develops in a woman's fifth to sixth decade of life [1]. The clinical presentation, symptoms, and complications show similarities to some other ovarian tumors. Such common symptoms are abdominal pain (54%), mass (31%), constipation, bleeding, weight loss, urinary frequency, and fever. MCT is generally a benign type of tumor; however some cases of malign transformation approximately occur in 1-2% of MCT cases [2]. The most common malignant transformation is squamous cell carcinoma (SCC) which is around 70–80% of malign transformation cases. Furthermore adenocarcinomatous, melanomas, carcinoid tumors, and different soft tissue sarcomas were reported [3–6]. The prognosis of the malign transformation of MCT depends on surgical stage; however it is extremely poor. We presented a case about this rare tumor, squamous cell carcinoma arising in a mature cystic teratoma with analyzing related literature.

## 2. Case 

**Figure 1:**
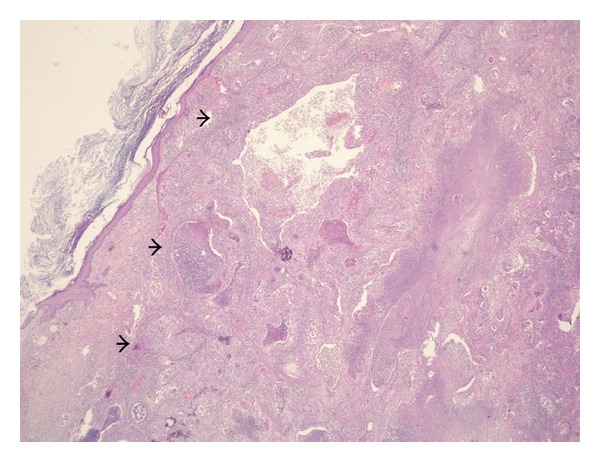
Sections from the mural nodule revealed moderately differentiated squamous cell carcinoma arising from the surface keratinizing squamous epithelium containing extensive necrotic areas (arrows). Hematoxylin and Eosin stain, ×2 objective.

**Figure 2:**
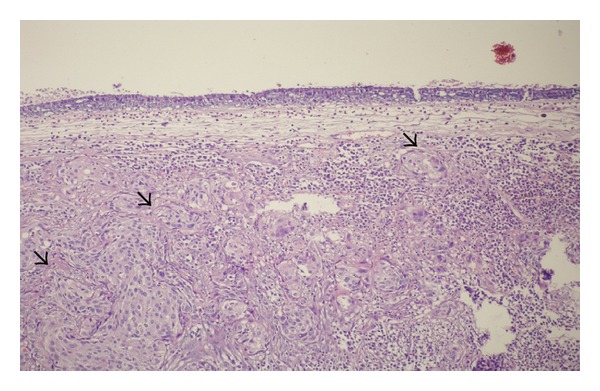
Another area lined by pseudostratified columnar epithelium with underlying squamous cell carcinoma (arrows). Hematoxylin and Eosin stain, ×4 objective.

A perimenopausal 52-year-old woman, gravida 3, para3, was admitted by our clinic in April 2012 with a one-month history of vaginal bleeding and progressive abdominal discomfort. She dose not have known medical history, medication, drinking alcohol problem, and family history of cancer. The patient's gynecologic examination and transvaginal ultrasound showed a mass in the right adnexa. Moreover, pelvic magnetic resonance imaging showed mass of about 7 cm in diameter in the right adnexal with coexisting cystic and solid densities. In the Doppler screening, there was a vascularization in solid component, and the value of the Resistance Index (RI) at Doppler scan was 0.71. Serum levels of cancer antigen are CA-125: 57 U/mL (<35). Colonoscopy was normal. Abdominal tomography and pelvic magnetic resonance imaging were negative for any metastasis. The patient underwent total abdominal hysterectomy with bilateral oophorectomy, omentectomy, pelvic lymph node dissection and debulking. Grossly an opened uniloculated cystic mass was received measuring 12 × 7.5 cm in size with 0.2 cm wall thickness. The cyst wall contained a mural nodule measuring 7 × 7 × 2 cm in size. Intraoperative pathology consultation revealed a malignant epithelial tumor consistent with squamous cell carcinoma arising in a mature cystic teratoma. According to the intraoperative pathology consultation, total abdominal hysterectomy, and bilateral salpingo-oophorectomy, omentectomy and pelvic lymph node dissection was made. In permanent sections the cyst wall showed features of a mature cystic teratoma that was composed of ectodermal elements. Sections from the mural nodule showed moderately differentiated squamous cell carcinoma arising from the surface epithelium. A final diagnosis of malignant transformation of moderately differentiated squamous cell carcinoma in a mature cystic teratoma with extensive necrosis was made (Figures 1 and 2). Omentum and the pelvic lymph nodes were free of tumor deposits. Ovarian surface was free of tumor and lymphovascular invasion was not identified. Consequently, tumor stage was determined as stage 1A. Two months after surgery six rounds carboplatin and paclitaxel were planned. Later the patient received second cycle treatment without any problem.

## 3. Discussion

Mature cystic teratomas (MCT), also called dermoid cysts, are the most common benign germ cell tumors of the ovary. Malignant transformation of MCT occurring within average frequency of 1%-2% is consistent with previous reports. Moreover, SCC consistent with previous reports and cases is the most diagnosed histologic type [7–9]. Tumor size, imaging characteristics and serum tumor markers are important risk factors for patients older than 45 years old for malignancy arising from MCT. Malignancy may occur at any age. It is observed in relatively postmenopausal stage. The median age of the diagnosis is between 45–60 years [1]. In the reported case above, the patient's age is 52 years old. The patient's age is consistent with the usual reported age range of this disease. Therefore, it is logical to have a higher suspicion of malignancy in MCT cases occurring in patients over the age of forty-five.

Abdominal pain followed by abdominal or pelvic mass is the most common symptoms. Incidentally, it could be observed with an asymptomatic adnexal mass detected on routine pelvic examination or pelvic imaging studies. In some other cases, various symptoms due to invasion of nearby organs are the presenting complaints, such as gastrointestinal symptoms of constipation or diarrhea, rectal bleeding, or urinary frequency [1]. In our case, the patient is presented with vaginal bleeding and abdominal distention. Tumor size should also be considered in order to predict malignancy, as Kikkawa et al. and Yamanaka et al. asserted that larger tumors with diameter 9.9 cm may be related to increasing risk of malignant transformation [8, 10]. In case we reported, the tumor size is 7 cm. 

Chiang et al. stated that higher concentration of serum CA-125 levels were associated with survival rates [9]. Prior relevant cases indicated that serum CA-125 antigen level is more applicable for other tumor markers in diagnosis [11]. In our case, CA-125 antigen was not measured due to technical impossibilities of routine clinical practice while serum CA-125 level preoperatively showed elevation.

Some imaging characteristics can be convenient determining malignant transformation. Clinical use of magnetic resonance imaging and computed tomography in preoperative diagnosis remains unclear. Most studies concluded that the Doppler detection method was a more useful indicator than serum SCC antigen levels [12, 13]. As we presented our case, vascularization in Doppler imaging, pointed out malignancy of the tumor.

The prognosis of this tumor is often reported to be very poor in the several previous studies. The prognosis heavily depends on the stage of the disease. Other factors effecting diagnosis are tumor grade, tumor dissemination, cyst-wall invasion, rupture, ascites, adhesion, growth pattern, and vascular invasion [3, 7].

The treatment modality choice to an ovarian MCT with malignant transformation is optimal debulking [4]. Chen et al. suggest that for the stage 1A diseas, can be treated by conservative surgery, either oophorocystectomy or oophorectomy. However unilateral salpingo-oophorectomy and surgical staging are proposed for young patients with fertility considerations with early-stage disease [4, 5]. According to Chen et al. stage 1A disease after surgical staging does not require adjuvant chemotherapy. Postoperative adjuvant therapy with the exception of alkylating agents in tumor stages later than 1A did not extend survival. Sakuma et al. supported in their case series to use of combination platinum/taxane chemotherapy. Postoperative radiotherapy could not obtain any success in previous reports in contrast to SCC of the uterine cervix [5]. Chen et al. asserted that this finding may be due to the fact that ovarian cancer with this type of histology has the same metastatic route as adenocarcinomas of the ovary [4]. Thus, after initial surgery, our patient was planned chemotherapy with carboplatin and paclitaxel. She received second cycle of the treatment without any complications.

## 4. Summary

Malignant transformation of MCT occurring within average frequency of 1%-2% is consistent with previous reports. The most common malign transformation is SCC which is around 70–80% of malign transformation cases. The prognosis of the malign transformation of MCT depends on surgery stage; however it is extremely poor. The patients should receive chemotherapy regardless of stage.
